# Editorial: Early embryonic development lineage

**DOI:** 10.3389/fcell.2026.1785662

**Published:** 2026-02-05

**Authors:** Lu Zhang, Shoulong Deng

**Affiliations:** State Key Laboratory of Animal Biotech Breeding, China Agricultural University, Beijing, China

**Keywords:** animal models, birth defects, embryonic development, gene function, single multi-omics

In recent years, the revolutionary progress in research methods, especially single-cell multi-omics, has ushered in a transformative era in the study of developmental biology. Modern developmental biology seeks to decipher the precise molecular, cellular, and metabolic programs that orchestrate the formation of complex organisms and the origin of related diseases. The seven articles in this collection exemplify this paradigm shift, offering a multi-scale view from gene function to tissue organization and linking fundamental molecular mechanisms directly to the origins of disease.

At the genetic level, in the article from Kolvenbach et al., we witness the pinpointing of specific causes of disease, as demonstrated by the identification of ABL1 haploinsufficiency as a monogenic cause of omphalocele via exome sequencing in a four-generation, multiplex family. This discovery underscores how single-gene perturbations can derail the entire morphogenetic and physiological function of the body. Similarly, the role of PPARGC1A in bovine embryo development highlights how master regulators of core cellular events, such as mitochondrial biogenesis, are essential for the start of very early life.

Turning to the tissue and organ scale, advanced research approaches are providing unprecedented resolution in elucidating related cellular networks. The construction of the first tissue-block-resolved transcriptomic atlas of the human fetal brainstem is a landmark resource by Liu et al. This study moves beyond bulk analysis to map the spatiotemporal expression of genes, directly linking developmental dynamics to vulnerability to neuropsychiatric disorders. Meanwhile, this kind of high-resolution approach is revolutionizing our understanding of complex birth defects, as reviewed for congenital heart disease (CHD) by Lv et al. The integration of single-cell and spatial multi-omics is dissecting CHD into a disorder of specific cardiac progenitor lineages and their molecular networks.

As the knowledge of developmental biology accumulates, practical approaches and potential therapies improve. Chen et al. present their work on enhancing pig SCNT embryo development via a sophisticated oviductal co-culture system, showing how mimicking the native microenvironment—through metabolic support and activation of pathways such as PI3K-AKT—can rescue developmental competence. In an avian model, the identification of a cocktail of cecal growth factors (GWEN) that promotes enteric neurosphere formation and colonization provides a blueprint for harnessing endogenous signaling to repair the enteric nervous system. In addition, the discovery of the intrinsic metabolic pattern and its role in the spatial organization of cell fate in human pluripotent stem cells is visualized by JC-1 (5,5,6,6’-tetrachloro-1’,1,3,3’ tetraethylbenzimi-dazoylcarbocyanine iodide) as a spatial tracker. This spatial dictation of cell fate reminds researchers that fundamental cell states, such as metabolism, are inseparable from the instructions for tissue patterning.

Collectively, these studies draw a coherent image: the path from a fertilized egg to a healthy individual is governed by an intricate hierarchy of genetic codes, molecular networks, metabolic dynamics, and cellular interactions. Dysfunctions or disruptions at any level can lead to birth defects or diseases. The future lies in continuing to integrate these scales—connecting genetic variants to specific cell compositions in developing tissues, and using that knowledge to create physiological models and specific interventions. This collection is a testament to the power of this integrated approach, charting a course toward a deeper understanding of life’s beginnings and opening new avenues for therapeutic methods.

